# Post-discharge surveillance and positive virus detection in two medical staff recovered from coronavirus disease 2019 (COVID-19), China, January to February 2020

**DOI:** 10.2807/1560-7917.ES.2020.25.10.2000191

**Published:** 2020-03-12

**Authors:** Yuanyuan Xing, Pingzheng Mo, Yu Xiao, Oiu Zhao, Yongxi Zhang, Fan Wang

**Affiliations:** 1 Department of Biological Repositories, Zhongnan Hospital of Wuhan University, Wuhan, China; 2 Department of Infectious Diseases, Zhongnan Hospital of Wuhan University, Wuhan, China; 3 These authors contributed equally to this study; 4 Department of Gastroenterology, Zhongnan Hospital of Wuhan University, Wuhan, China

**Keywords:** COVID-19, post-discharge surveillance, positive detection, medical staff

## Abstract

Since December 2019, 62 medical staff of Zhongnan Hospital in Wuhan, China have been hospitalised with coronavirus disease 2019. During the post-discharge surveillance after clinical recovery, swabs were positive in two asymptomatic cases (3.23%). Case 1 had presented typical clinical and radiological manifestations on admission, while manifestation in Case 2 was very mild. In conclusion, a small proportion of recovered patients may test positive after discharge, and post-discharge surveillance and isolation need to be strengthened.

Since early December 2019, several pneumonia cases of unknown aetiology have occurred in Wuhan and rapidly spread throughout China [1,2]. The International Committee on Taxonomy of Viruses (ICTV) has named this virus SARS-CoV-2, and it belongs to the same species as SARS-CoV [3]. Meanwhile, the World Health Organization (WHO) has named the virus-infected pneumonia coronavirus disease 2019 (COVID-19) [4]. As at 5 March 2020, a total of 80,409 COVID-19 cases and 3,012 deaths (3.75%) have been reported in mainland China [5]. The 52,045 recovered cases (64.73%) were further quarantined at home for at least 2 weeks [5]. However, potential infectivity of these recovered cases was still unclear. Thus, we implemented consecutive virus surveillance among medical staff recovered from COVID-19 at our hospital and aimed to investigate their potential infectivity after discharge.

## Case follow-up

Since January 2020, 62 medical staff of Zhongnan Hospital of Wuhan University have been diagnosed with COVID-19 by detecting SARS-CoV-2 nucleic acid in throat swab samples according to the manufacturer’s protocol (Shanghai BioGerm Medical Technology, Shanghai, China). Briefly, the RT-PCR assay for SARS-CoV-2 amplifies simultaneously two target genes: open reading frame 1ab (ORF1ab) and the ORF for the nucleocapsid protein (N). Target 1 (ORF1ab): forward primer CCCTGTGGGTTTTACACTTAA; reverse primer ACGATTGTGCATCAGCTGA; probe 5’-VIC-CCGTCTGCGGTATGTGGAAAGGTTATGG-BHQ1-3’. Target 2 (N): forward primer GGGGAACTTCTCCTGCTAGAAT; reverse primer CAGACATTTTGCTCTCAAGCTG; probe 5’-FAM- TTGCTGCTGCTTGACAGATT-TAMRA-3’. Positive (pseudovirus with a fragment of ORF1ab and N) and negative (pseudovirus with a standard fragment) quality control samples were tested simultaneously. A cycle threshold (Ct) value of less than 37 was defined a positive test, while a Ct value of more than 40 was defined as a negative test. For the cases with an intermediate Ct value (37–40), a second sample was tested and weakly positive was defined as a recurrence of Ct value of 37–40. The diagnostic criteria were based on the recommendation from the National Institute for Viral Disease Control and Prevention (China) [6].

All confirmed cases were hospitalised and isolated for treatment. The discharge criteria were: (i) afebrile for at least 3 days, (ii) obvious alleviation of respiratory symptoms, (iii) improvement in radiological abnormalities on chest computed tomography (CT) or X-ray and (iv) two consecutive negative detections of SARS-CoV-2 at least 24 h apart [7]. After discharge, all cases were kept under surveillance and quarantined at home for at least 14 days; all cases had a throat swab test for SARS-CoV-2 every day or every other day at least 5 times. For those with positive virus detection during this period, we extracted and analysed the medical records.

This study was approved by the ethics committee of Zhongnan Hospital of Wuhan University (Number 2020011), and written informed consent was obtained from patients.

### Case 1

**Figure 1:**
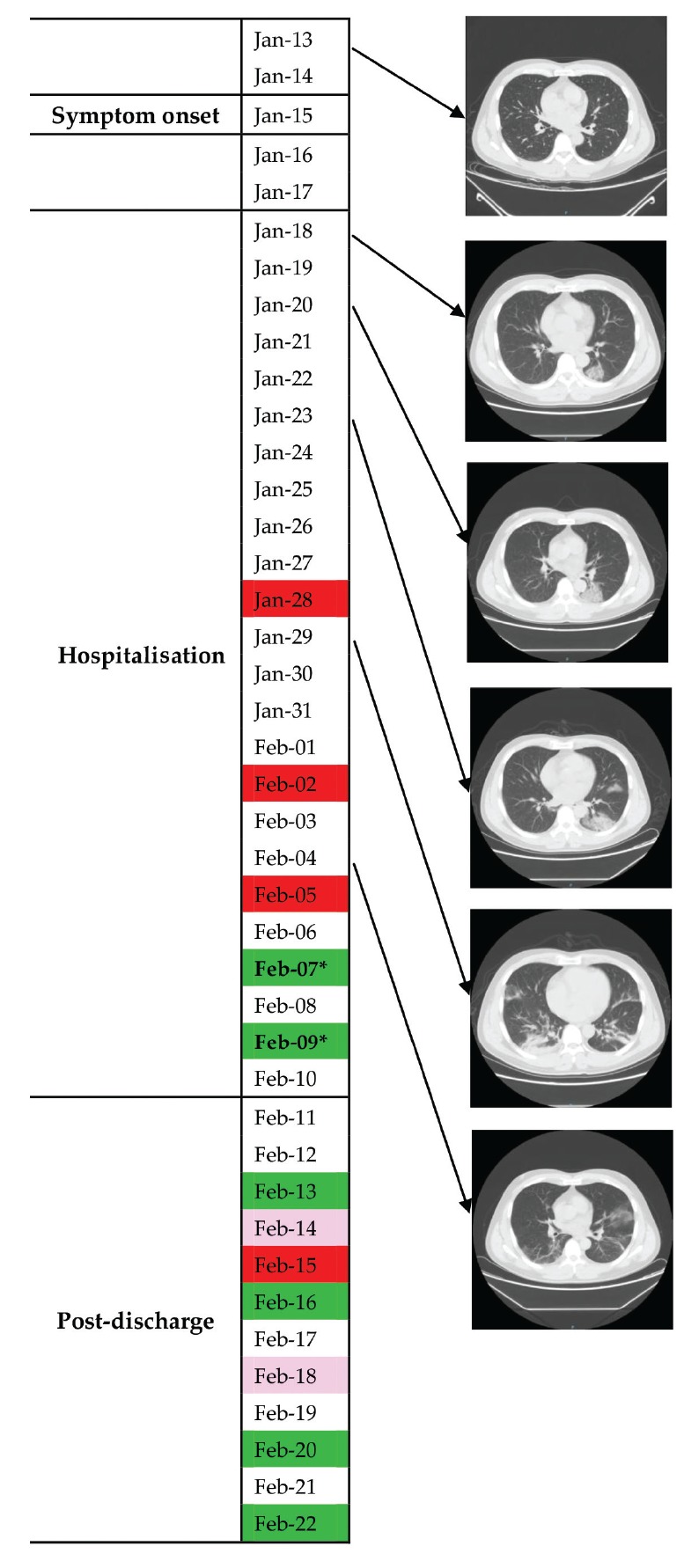
Throat swab virus tests and chest computed tomography findings of COVID-19 Case 1 from symptom onset to post-discharge, China, January–February 2020

Case 1 was a male doctor in his 40s with an exposure history to COVID-19 patients. He experienced fever (up to 39.3 °C), chill and fatigue on 15 January (Figure 1). Chest CT showed lung infection in the lower left lobe on 18 January, and he was admitted to hospital on the same day. During the hospitalisation from 18 January to 10 February, his condition first deteriorated and then reached remission on 28 January. Throat swab tests for SARS-CoV-2 were positive on 28 January and 2 February, and turned negative on 7 and 9 February. Stool tests of SARS-CoV-2, first conducted on 7 February, were also negative on 7 and 9 February. The exact Ct values were unavailable.

Chest CT showed worse status on 18, 20, 23 and 29 January and showed improved status on 4 February.

Red: positive virus detection in throat swabs; pink: weakly positive virus detection in throat swabs; green: negative virus detection in throat swabs; dates marked with an asterisk: negative virus detection in stool.

After discharge on 10 February, he was kept under surveillance and quarantined at home. He did not experience discomfort during the follow-up period. The results of consecutive throat swab tests were negative on 13 February, weakly positive on 14 February, positive on 15 February, negative on 16 February, weakly positive on 18 February, negative on 20 February and negative on 22 February. Stool was not tested after discharge.

### Case 2

**Figure 2:**
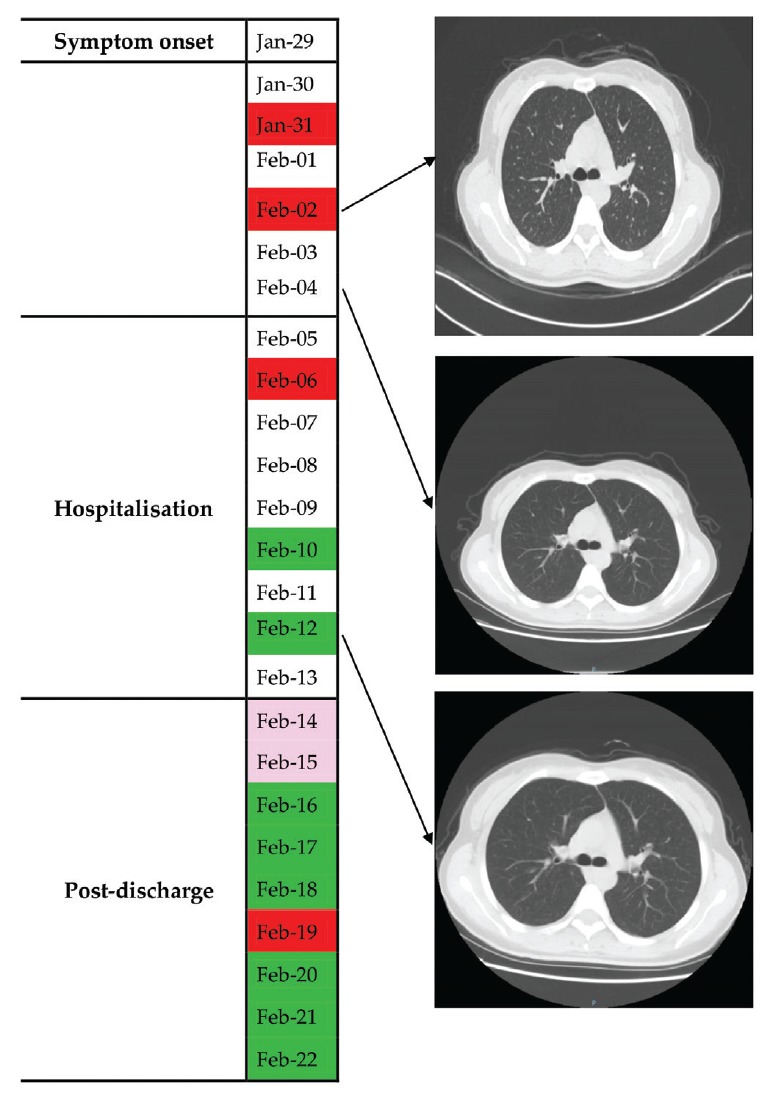
Throat swab virus tests and chest computed tomography findings in COVID-19 Case 2 from symptom onset to post-discharge, China, January–February 2020

Case 2 was a female nurse in her 20s. She experienced headache and pharyngalgia but no fever on 29 January (Figure 2). Throat swab tests for SARS-CoV-2 were positive on 31 January and 2 February. However, chest CT showed no abnormalities on 2 February. This patient was admitted to hospital on 5 February. Throat swab test remained positive on 6 February and turned negative on 10 and 12 February. Stool was not tested in Case 2.

Chest CT showed no abnormalities on 2, 4 and 12 February. Red: positive virus detection in throat swabs; pink: weakly positive virus detection in throat swabs; green: negative virus detection in throat swabs.

After discharge on 13 February, Case 2 was kept under surveillance and quarantined at home. She did not experience discomfort during the follow-up. The results of consecutive throat swab tests were weakly positive on 14 and 15 February, negative on 16, 17 and 18 February, positive on 19 February and negative on 20, 21 and 22 February.

## Discussion

On 21 February 2020, a previously recovered COVID-19 patient in Chengdu (Sichuan province, China) was re-hospitalised after testing positive for the virus [8]. This aroused our great concern regarding potential infectivity of the recovered patients, especially for those asymptomatic cases with positive virus detection after discharge.

In our study, we conducted surveillance by regular virus testing and detected two positive cases (3.23%) among the 62 recovered medical staff. Surprisingly, Case 1 showed typical clinical and radiological manifestations on admission, while the manifestation in Case 2 was not typical. Moreover, both throat swab and stool tests turned negative in Case 1 before discharge. During home isolation, none of the two cases experienced discomfort, indicating that disease relapse was unlikely.

Currently, it is difficult to find a reasonable explanation for these observations. It was assumed that the virus can easily be detected in the upper respiratory tract at the stage of early infection. When the disease progressed, the virus was more likely to appear in the lower respiratory tract and other locations such as intestines and blood [9]. Thus, the virus may not be detected in throat swabs, especially for cases without expectoration. This may explain why some patients had negative RT-PCR for SARS-CoV-2 at initial presentation despite positive findings in chest CT, or positive RT-PCT and negative CT findings at initial presentation [10,11]. 

Moreover, our findings seem to indicate that after hospitalised treatment, there could be a possibility that a small proportion of clinically recovered patients may still carry a small amount of virus which is hard to detect. The current standard for diagnosing COVID19, the RT-PCR-based method, showed a high accuracy of 97% and the specific primers and probes guaranteed its diagnostic specificity, although 3% of cases may test false-negative because of potential sampling error [11]. Both cases had a positive detection (including weakly positive) three times during the follow-up, which decreased the possibility of false positives in these two cases.

After fulfilling the Chinese current criteria for discharge, it may still take a few days for the immune system to completely eliminate the residual viruses in the body [7]. During this period, the virus may rebound and test positive, but the patients were asymptomatic and chest CT showed no deterioration. If the patients’ immunity decreases, there is a risk of a relapse. On 6 February 2020, a recovered COVID-19 patient in Changde (Hunan province, China) had a fever and cough 2 days after discharge, and chest CT showed worsened status [12].

## Conclusion

A small proportion of recovered patients may have positive virus detection after discharge, and the positivity does not necessarily mean that the patient is transmissive. These findings need further investigation and thus post-discharge surveillance is suggested.
